# The Systemic Administration of the Histamine H_1_ Receptor Antagonist/Inverse Agonist Chlorpheniramine to Pregnant Rats Impairs the Development of Nigro-Striatal Dopaminergic Neurons

**DOI:** 10.3389/fnins.2019.00360

**Published:** 2019-04-16

**Authors:** Berenice Márquez-Valadez, Guillermo Aquino-Miranda, Mijail-Oliver Quintero-Romero, Helena Papacostas-Quintanilla, Antonio Bueno-Nava, Carolina López-Rubalcava, Néstor Fabián Díaz, José-Antonio Arias-Montaño, Anayansi Molina-Hernández

**Affiliations:** ^1^Departamento de Fisiología, Biofísica y Neurociencias, Centro de Investigación y de Estudios Avanzados del Instituto Politécnico Nacional, Mexico City, Mexico; ^2^Laboratorio de Investigación en Células Troncales y Biología del Desarrollo, Departamento de Fisiología y Desarrollo Celular, Instituto Nacional de Perinatología Isidro Espinosa de los Reyes, Mexico City, Mexico; ^3^Laboratorio de Psicofarmacología y Trastornos de la Alimentación, Departamento de Farmacobiología, Centro de Investigación y de Estudios Avanzados delInstituto Politécnico Nacional, Mexico City, Mexico; ^4^División de Neurociencias, Instituto Nacional de Rehabilitación Luis Guillermo Ibarra Ibarra, Mexico City, Mexico

**Keywords:** histamine, H_1_ receptor, chlorpheniramine, substantia nigra *pars compacta* development, dopaminergic neurons, motor activity

## Abstract

The dopaminergic and histaminergic systems are the first to appear during the development of the nervous system. Through the activation of H_1_ receptors (H_1_Rs), histamine increases neurogenesis of the cortical deep layers, while reducing the dopaminergic phenotype (cells immunoreactive to tyrosine hydroxylase, TH^+^) in embryo ventral mesencephalon. Although the function of histamine in neuronal differentiation has been studied, the role of H_1_Rs in neurogenesis has not been addressed. For this purpose, the H_1_R antagonist/inverse agonist chlorpheniramine was systemically administered (5 mg/kg, i.p.) to pregnant Wistar rats (gestational days 12–14, E12–14), and control and experimental embryos (E14 and E16) and pups (21-day-old) were evaluated for changes in nigro-striatal development. Western blot and immunohistochemistry determinations showed a significant increase in the dopaminergic markers’ TH and PITX3 in embryos from chlorpheniramine-treated rats at E16. Unexpectedly, 21-day-old pups from the chlorpheniramine-treated group, showed a significant reduction in TH immunoreactivity in the substantia nigra *pars compacta* and dorsal striatum. Furthermore, striatal dopamine content, evoked [^3^H]-dopamine release and methamphetamine-stimulated motor activity were significantly lower compared to the control group. These results indicate that H_1_R blockade at E14–E16 favors the differentiation of dopaminergic neurons, but hampers their migration, leading to a decrease in dopaminergic innervation of the striatum in post-natal life.

## Introduction

The commitment and maturation of dopaminergic neurons is a complex process coordinated by secreted molecules (mainly sonic hedgehog and fibroblast growth factor 8) and the expression of transcription factors, which are finely coordinated in a time and spatial manner (Hegarty et al., 2013). The specification of these processes begins at embryo day 10.5 (E10.5) in the rat vMes, and committed dopaminergic neurons can be identified by the expression of the transcription factors Lim-homeodomain 1A and 1B (LMX1A and LMX1B), and Forkhead box A1/2 (FOXA1/2) (Smidt et al., 2000; Andersson et al., 2006; Guerrero-Flores et al., 2017). These transcription factors are no longer expressed by mature dopaminergic neurons, which instead express TH (the rate-limiting enzyme of dopamine biosynthesis), the paired-like homeodomain transcription factor 3 (PITX3), and the nuclear orphan receptor 1 (NURR1) (Lauder and Bloom, 1974; Specht et al., 1981a,b; Smidt et al., 2004; Yan et al., 2011).

When neural progenitors (NPCs) are specified to the dopaminergic phenotype, they migrate from the ventricular zone to the intermediate zone to originate the midbrain dopaminergic nuclei, namely SNpc (A9 nucleus), ventral tegmental area (VTA; A10 nucleus), and retrorubral field (A8 nucleus) (Vitalis et al., 2005). Upon arrival to their neuroanatomical target, dopaminergic neurons start to extend their axonal processes dorsally, through the mesencephalic flexure (starting at E13) and the immature medial forebrain bundle, and at E14.5 the axons arrive to the striatum, where TH^+^ fibers gradually increase (Specht et al., 1981b; Gates et al., 2004). In the rat, the complete maturation and survival of dopaminergic neurons occurs at postnatal day 21, P21 (Oo and Burke, 1997).

During the development of the nervous system, the transitory fetal histaminergic system co-localizes with serotonin positive neurons, which will originate the raphe nuclei in the adult brain. Neurons immunoreactive to histamine are detected at E12 in the rat mesencephalon/rhombencephalon and histamine levels reach their maximum at E14 (Vanhala et al., 1994). Histamine actions are mediated by four G protein-coupled receptors, H_1_–H_4_ (Panula et al., 2015), and H_1_ and H_2_ receptors are widely distributed in the central nervous system (CNS) at E14 and E15, respectively (Kinnunen et al., 1998; Heron et al., 2001; Karlstedt et al., 2001; Solís et al., 2017).

By activating H_1_ receptors (H_1_Rs), histamine promotes neuron differentiation from cerebral cortex and vMes stem cells (NSCs) at E14 and E12, respectively, as well as from adult NSCs *in vitro* (Molina-Hernández and Velasco, 2008; Bernardino et al., 2012; Escobedo-Ávila et al., 2014). In cortical NSCs, histamine favors their differentiation into deep layer FOXP2-positive neurons, while in vMes NSCs the amine impairs the differentiation of neurons immunoreactive to TH, a marker of catecholaminergic neurons. The effects observed in cortical and mesencephalic NSCs are prevented by the H_1_R antagonist/inverse agonist chlorpheniramine (Molina-Hernández et al., 2013; Escobedo-Ávila et al., 2014).

The relevance of histamine and H_1_Rs in development is highlighted by knockout mouse models, in which knocking out the expression of either H_1_Rs or histidine decarboxylase (the limiting histamine-producing enzyme) leads to impairment of brain functions such as learning and memory, wakefulness and nociception (Mobarakeh et al., 2005; Chepkova et al., 2012; Ambree et al., 2014; Parmentier et al., 2016). Although information on the role of histamine in neuronal differentiation has recently increased, the postnatal consequences of H_1_R blockade during neurogenesis have not been addressed.

Therefore, to study the participation of the H_1_R in the development of the dopaminergic nigro-striatal system, we analyzed H_1_R expression at E12 and E14 in the vMes, blocked it pharmacologically by systematically injecting pregnant rats with chlorpheniramine (5 mg/kg, i.p.) on days E12, E13, and E14, and assessed the effect at E14 and E16 (dopaminergic differentiation and maturation) and in post-natal day 21 offsprings (P21, mature dopaminergic system).

## Materials and Methods

### Animals

All experiments and animal manipulation were in accordance to the Animal Welfare Assurance (A5281-01), “Guide for the Care and Use of Laboratory Animals” (NIH Publication No. 80-23, revised 1978) and “Norma Official Mexicana para la Producción, Cuidado y Uso de Animales de Laboratorio” (NOM-062-ZOO-1999). The protocol was approved by the Cinvestav Animal Care Committee and the Instituto Nacional de Perinatología Isidro Espinosa de los Reyes Research Animal Care, Biosecurity, and Ethic committees (protocol 3230-21202-01-2015).

Female Wistar rats (250–300 g) were bred and mated in the Cinvestav facilities. The morning after mating was established as E0.5. Twenty-four hours later pregnant rats were transported to the Instituto Nacional de Perinatología animal facilities, housed individually and maintained in standard conditions (12:12 h light/dark cycle, 21 ± 2°C and 40% relative humidity) with free access to food and water.

**FIGURE 1:**
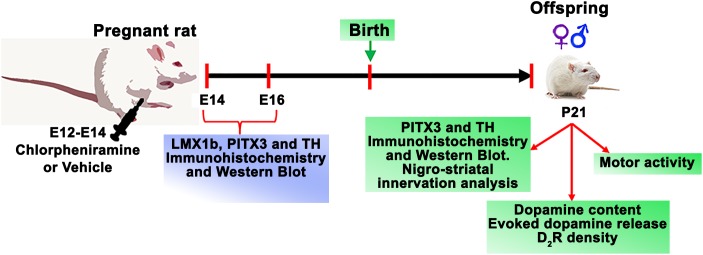
Time line for experimental procedures.

The number of pregnant rats were calculated as recommended by Rojas-Amigo (2014). Pregnant rats were injected daily (E12, E13, and E14) with vehicle (injectable water) or chlorpheniramine (5 mg/kg/day, i.p.; Sigma-Aldrich, St. Louis, MO, United States) per experiment (Figure 1). To obtain embryo tissues, animals were decapitated, and the embryos were rapidly recovered by cesarean at E14 and E16. The vMes was dissected under a stereoscopic microscope (Olympus SZX16, Shinjuku, Tokyo, Japan) or fixed by immersion in Boüin’s solution and then frozen to obtain coronal slices (10 μm thick) in a cryostat (Leica CM1850, Wetzlar, Germany).

To study changes in the nigro-striatal dopaminergic system, P21 pups (males and females, Figure 1) obtained from control and chlorpheniramine-treated pregnant rats, were randomly assigned to neurochemical assays (dopamine content, dopamine D_2_ receptor density, and dopamine release), immunodetection (Western blot and immunofluorescence), and evaluation of motor activity. For dopamine content and release, receptor density, and Western blot, animals were decapitated and brains were rapidly removed from the skull and SNpc and striatum tissues were obtained as described below. For immunofluorescence, animals were anesthetized with pentobarbital (50 mg/kg, i.p.; Laboratorios PiSA, Mexico City, Mexico) and intracardially perfused with Boüin’s solution at a constant flow rate of 0.6 ml/min (Masterflex L/S Digital Miniflex Pump, Vernon Hills, IL, United States). Afterward, the brain was extracted and frozen to obtain coronal slices (30 μm thick).

### RNA Extraction and End-Point RT-PCR

H_1_R expression at E12 and E14 was determined in a vMes pool from 6 (E12) or 4 (E14) embryos obtained from eight different control pregnant rats. Total RNA was isolated using TRIZOL reagent (Invitrogen, CA, United States). One microgram RNA was reverse transcribed with 0.5 μg Oligo(dT), 1 mM dNTPs, 1 U RNAsin Ribonuclease Inhibitor and 5 U AMV Reverse Transcriptase (Promega, Madison, WI, United States). For PCR analysis, 500 μg cDNA were assayed in a mix containing 20 pmol of specific primers (Integrated DNA Technologies, Skokie, IL, United States), 0.4 mM dNTPs, 2 mM MgCI_2_, and 1.25 U GoTaq DNA polymerase (Promega). The primers used for the amplification of H_1_R and GAPDH (internal control) were (Solís et al., 2017): H_1_R, Forward: 5′-CTTCTACCTCCCCACTTTGCT-3′, Reverse: 5′-TTCCCTTTCCCCCTCTTG-3′; GAPDH, Forward: 5′-GGACCTCATGGCCTACATGG-3′, Reverse: 5′-CCCCTCCTGTTGTTATGGGG-3′.

The amplifying conditions were as follows: denaturalization at 95°C for 15 min followed by 30 cycles of denaturalization at 95°C for 30 s, annealing at 56°C (H_1_R) or 58°C (GAPDH) for 30 s, and elongation at 72°C for 30 s, with final extension at 74°C for 10 min. Adult cerebral cortex was used as a positive control and total RNA as negative control. The semi-quantitative analysis was performed using ImageJ software^1^, on the specific bands stained with GelRed (Biotium, Hayrward, CA, United States) observed after electrophoresis in 2% agarose. Representative bands were purified and sequenced at Unidad de Biología Molecular, Instituto de Fisiología Celular, Universidad Nacional Autónoma de México (Mexico City, Mexico).

### Immunohistochemistry

Two fixed embryos from three different pregnant rats (three experiments) per group were used (E14 and E16). On the other hand, one female and male were obtained from four different litters, for a total of eight offspring from four independent experiments at P21. The quantitative immunostaining and TH-positive cells analysis was performed at P21 with four consecutive slices taken between Bregma -5.6 to -5.8 mm (Paxinos and Watson, 2013).

Slices from embryos or brains (P21 animals) were washed with PBS containing 0.1% albumin, blocked and permeabilized (10% normal goat serum, NGS, and 0.3% Triton-X100 in PBS), and incubated overnight at 4°C with the following primary antibodies: rabbit polyclonal anti-H_1_R (1:250, **RRID:AB_2277328**, Santa Cruz Biotechnology, Dallas, TX, United States), anti-LMX1B (1:200, **RRID:AB_1269316**, Abcam, Cambridge, MA, United States), anti-PITX3 (1:200, **RRID:AB_2165300**, Abcam) and anti-TH (1:200, **RRID:AB_297840**, Abcam), and mouse monoclonal anti-NES (Nestin; 1:200, **RRID:AB_370407**, GeneTex, Irvine, CA, United States), anti-TUJ1 (1:2000, **RRID:AB_2210524**, Merck Millipore, Burlington, MA, United States) and anti-MAP2 (1:500, RRID:AB_369978, GeneTex). After washing with PBS, slices were incubated for 1 h with the following fluorescent secondary antibodies: Alexa Fluor 488 anti-rabbit IgG (1:1000, **RRID:AB_143165**, Thermo Fisher Scientific, Waltham, MA, United States), Alexa Fluor 568 anti-mouse IgG (1:1000, **RRID:AB_2534072**, Thermo Fisher Scientific) or IRDye 800CW donkey anti-rabbit (1:1000, **RRID:AB_621848**, LI-COR Bioscience, Lincoln, NE, United States). Nuclei were stained with DAPI (5 ng/ml, Sigma-Aldrich) or DRAQ5^TM^ (5 μM, Abcam) as internal controls and preparations were mounted in Aqua PolyMount (Polysciences, Warrington, PA, United States). For negative controls primary antibodies were omitted.

Microphotographs were obtained using an epifluorescence microscope (Olympus IX81) with a charge-coupled device camera (ORCA-Flash 2.8 CCD model C11440 Hamamatsu; Hamamatsu, Honshu, Japan). For the quantitative analysis of TH signals in brain slices (P21 animals), slices were scanned in the Odyssey CLx Imaging system at 800 nm and the fluorescence was evaluated using the Image Studio 4.0 software (LI-COR Bioscience). Images were processed with Adobe Photoshop CS6 (San Jose, CA, United States).

### Western Blot

For Western blot analysis, a pool of vMes tissue from one litter at E14 and E16, or the bilateral P21 SNpc from one male and one female were used per experiment for a total of four independent experiments per group. The tissue was homogenated in lysis buffer (25 mM Tris–HCl, pH 7.4, 1% IGEPAL, 100 mM NaCI) containing a protease inhibitor cocktail (Amresco Inc., Solon, OH, United States) and centrifuged (13,000 ×*g* at 4°C for 10 min). The supernatant was recovered and the protein was quantified by the Bradford assay (Bradford, 1976).

Samples (30 μg of total protein) or 3 μl of a commercial molecular weight standard (26634, Spectra^TM^ Multicolor Broad Range Protein Ladder, Thermo Fisher Scientific or GTX50875, Trident Prestained Protein Ladder, GeneTex) were loaded in denaturalizing 10% poly-acrylamide gels and electrophoresis was performed using the Mini-Protean II system (Bio-Rad, Hercules, CA, United States). Proteins were transferred to nitrocellulose membranes (Santa Cruz Biotechnology), using the Trans-Blot^®^ semi-dry transfer cell system (Bio-Rad) as previously described (Villanueva, 2008), and incubated overnight at 4°C with the primary rabbit polyclonal antibodies anti-LMX1B (1:500, Abcam), anti-PITX3 (1:500, Abcam) or anti-TH (1:500, Abcam), and mouse monoclonal anti-GADPH (internal control; 1:1500, GeneTex). Secondary infrared donkey antibodies (1:10,000 dilution) were: IRDye 800CW anti-rabbit, IRDye 800RD anti-mouse, IRDye 680RD anti-mouse (**RRID:AB_10953628**), IRDye 680RD anti-rabbit (**RRID:AB_10954442**; LI-COR Bioscience). Membranes were scanned in the Odyssey CLx system and the fluorescence was analyzed with Image Studio ver.4.0 software (LI-COR Bioscience).

Tissue from liver or spleen were used as negative controls to anti-LMX1B and anti-PITX3, respectively (Supplementary Figure S1).

### Radioligand Binding Assays

To determine H_1_R density at E12 (three litters per experiment) and E14 (one litter per experiment), a total membrane preparation for five independent experiments per group were obtained combining the telencephalon and mesencephalon. The tissue was homogenized in a hypotonic solution (10 mM Tris–HCI, 1 mM EGTA, pH 7.4) and centrifuged (20,000 ×*g* at 4°C for 20 min). The resulting pellet (membranes) was re-suspended in 450 μl incubation buffer (50 mM Tris–HCI, pH 7.4) and sonicated (3×, 5 s). Binding assays were performed as previously in detail elsewhere (Soria-Jasso et al., 1997). Briefly, membrane aliquots were incubated for 60 min at 30°C in 100 μl incubation buffer containing a nearly saturating concentration (10 nM) of the selective H_1_R antagonist [^3^H]-mepyramine (PerkinElmer, Waltham, MA, United States). Specific binding was defined as that insensitive to 10 μM unlabelled mepyramine and was normalized to the amount of protein per sample determined by the BCA method (E12 ∼2 μg; E14 ∼22 μg).

The density of dopamine D_2_ receptors in striatal nerve terminals was evaluated in a synaptosomal membrane preparation obtained as described in detail elsewhere (Márquez-Gómez et al., 2018) from a pool of P21 males or females (four pups per determination for a total of three experiments per group). Synaptosomes were lysed in a hypotonic solution (10 mM Tris–HCI, 1 mM EGTA, pH 7.4), the suspension was centrifuged (20,000 ×*g*, 4°C, 20 min) and the pellet (membranes) was re-suspended in 0.5 ml incubation buffer (50 mM Tris–HCI, 10 mM KCl, 4 mM MgCl_2_, 1 mM EDTA, 100 μM ketanserin, pH 7.4). Aliquots were incubated with 2 nM [^3^H]-spiperone (PerkinElmer) in a final 100 μl volume at 25°C for 90 min. Non-specific binding was defined in the presence of 100 μM (±)-butaclamol (Sigma-Aldrich).

The incubation was stopped by filtration through Whatman GF/B glass microfiber filters (GE Healthcare Life Science, Marlborough, MA, United States), pre-soaked for 2 h in 0.3% polyethylenimine and the radioactivity retained in the filters was determined by scintillation counting. Data were normalized to the amount of protein per sample (∼72 μg).

### High-Performance Liquid Chromatography Coupled to Electrochemical Detection (HPLC-EC)

Both striata of one P21 pup per gender and group were dissected for a total number of independent experiments of four for the males or seven (control) and six (chlorpheniramine-treated) for the females, placed in a solution of 0.4 M HClO_4_ containing 5 mM EGTA and 2.5 mM Na_2_S_2_0_5_ and homogenized. Homogenates were centrifuged (13,000 ×*g*, 4°C, 15 min), the supernatant was recovered, passed through a 0.22 μm Acrodisc filter (Pall Corporation, Port Washington, NY, United States) and 50 μl were used to determine the dopamine concentration by HPLC-EC using an Alltech HPLC pump (model 626, Grace Discovery Sciences, Columbia, MD, United States) coupled to an electrochemical detector (ESA, Coulochem III, Chelmsford, MA, United States). A 100 mm × 2 mm analytical column with a particle size of 3 μm (Microbore, BASi, West Lafayette, IN, United States) was used and the mobile phase (flow rate 0.3 ml/min) consisted of 29 mM monobasic phosphate pH 3.0, 3.5 mM sodium octyl-sulfate, 0.43 mM EDTA, 0.6 mM tetrahydrofuran and methanol (3.75% v/v; Sigma-Aldrich). The detection settings were: guard cell +350 mV (ESA 5020) and analytical cell (ESA 5911A) potentials *E1* +200 mV and *E2* -200 mV. The peak signal was automatically delivered to a computer using the program EZChrom SI (version 3.2.1, Agilent Technologies, Santa Clara, CA, United States). The dopamine concentration was estimated by interpolating the experimental data to a standard curve obtained from five known dopamine concentrations (5–80 nM). Data were normalized to the amount of protein per sample (∼10 μg of sample).

### [^3^H]-Dopamine Release From Striatal Slices

A pool of cross-chopped P21 striatal slices (250 μm × 250 μm) were obtained from four males and four females’ pups, for a total of four or six independent experiments, respectively, per group. Slices were placed in ice-cold Krebs-Henseleit (KH) solution (126 mM NaCI, 3 mM KCI, 1 mM MgSO_4_, 1.2 mM KH_2_PO_4_, 25 mM NaHCO_3_, 11 mM D-glucose; pH 7.4 after saturation with O_2_/CO_2_, 95:5% v:v) without CaCl_2_ to reduce excitotoxicity. Thereafter, slices were equilibrated in KH solution containing 1.8 mM CaCl_2_ for 30 min at 37°C, changing the solution every 10 min. The slices were then transferred to 1 ml KH solution containing 50 nM [^3^H]-dopamine (PerkinElmer), the monoamine oxidase inhibitor pargyline (10 μM) and the antioxidant ascorbic acid (200 μM) to prevent [^3^H]-dopamine degradation, and 1 μM desipramine and 1 μM fluoxetine to minimize [^3^H]-dopamine uptake by noradrenergic and serotoninergic terminals, respectively.

After 30 min at 37°C, the slices were washed with KH solution, randomly distributed in perfusion chambers of a superfusion system (Brandel 2500, Gaithersburg, MD, United States), and perfused (1 ml/min) with KH solution supplemented with pargyline and ascorbic acid to minimize [^3^H]-dopamine degradation and 1 μM GBR-12909 (Sigma-Aldrich) to prevent [^3^H]-dopamine reuptake by dopaminergic terminals. After 20 min, two basal fractions were collected (1 ml each) and at the third fraction [^3^H]-dopamine release was evoked by switching for 1 min to KH solution containing 30 mM KCl (equimolarly substituted for NaCl). The perfusion was returned to normal KH solution and a further seven fractions were collected. The Ca^2+^-dependence of [^3^H]-dopamine release was tested in KH solution to which no CaCI_2_ was added.

The tritium content in the perfusion fractions was determined by scintillation counting, and the tritium remaining in the tissue from each chamber was determined after treating the tissue with 1 ml HCl (1 M) for 2 h before adding scintillator liquid. All fractional values were normalized to the fraction collected immediately before switching to the depolarizing solution and the area under the curve was obtained to calculate the total [^3^H]-dopamine release.

### Open Field Test

Pups (P21) from control and chlorpheniramine-treated animals were randomly selected. Motor activity was tested under basal conditions or following the injection of the dopamine-releasing agent methamphetamine (1 mg/kg; i.p.). Thirty minutes after the injection, pups were placed individually at the corner of an open-field maze (44 cm × 45 cm × 36 cm) divided into 12 quadrants, and left to freely explore for 5 min under red light. An infrared actimeter (Letica model LE 8811, PanLab, Cornellà, BARC, Spain) and Actitrack software 2.0 (Coulbourn Instruments, Holliston, MA, United States) were used to evaluate total square crosses, speed, and distance. The box was carefully cleaned between tests.

Pups were obtained from four independent experiments. A total of 15 control and 16 chlorpheniramine-treated males, and, 21 control and 23 chlorpheniramine-treated females were used. Rats from control and chlorpheniramine groups were further divided into basal (male: control, *n* = 8 and chlorpheniramine, *n* = 9; females: control, *n* = 9 and chlorpheniramine, *n* = 10) and methamphetamine-treated (male: control, *n* = 7 and chlorpheniramine, *n* = 7; females: control, *n* = 12 and chlorpheniramine, *n* = 13) groups, for basal and dopamine stimulate motor activity.

### Statistics

Graphs and statistics were performed using the GraphPad Prism 6.0 software (GraphPad Software, La Jolla, CA, United States). All data are presented as means and the associated standard error (SEM) expressed as the percentage of change of the control group, except for the motor activity analysis were data are the means (SEM) from absolute values. Data from control and chlorpheniramine groups were compared with one-tailed unpaired Student’s *t*-test and differences were considered significant at *P* < 0.05.

## Results

### H_1_R Expression at E12 and E14

**FIGURE 2:**
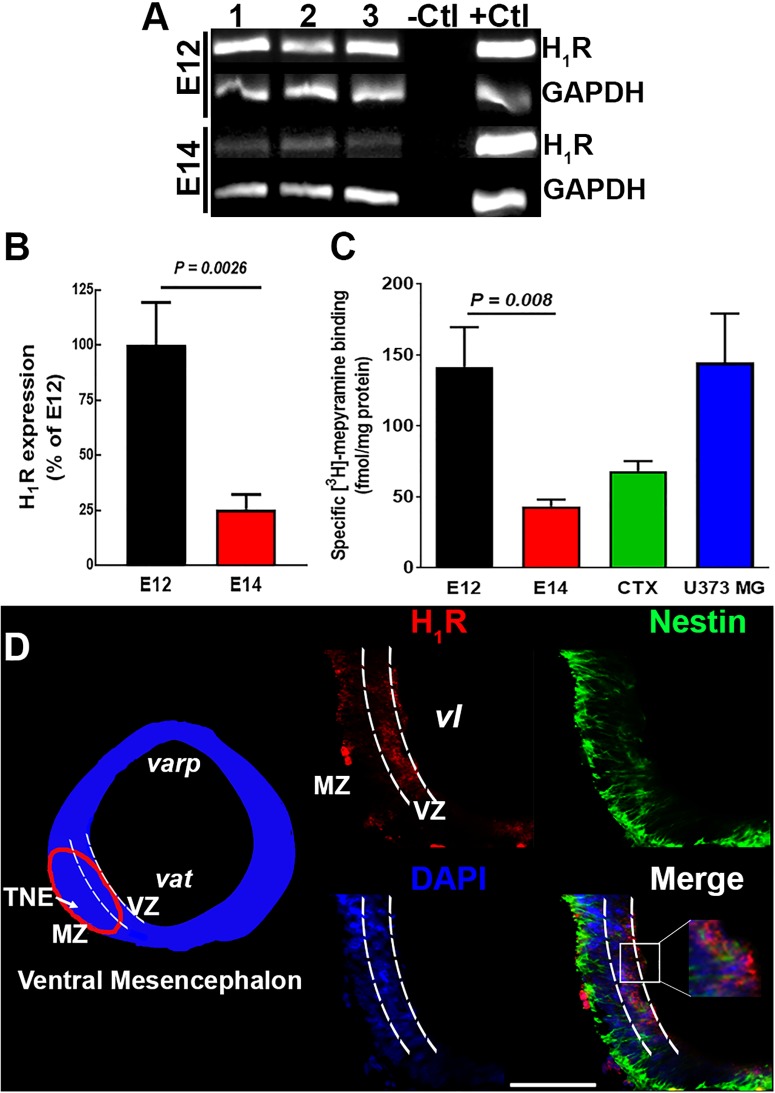
H_1_R expression in the ventral mesencephalon of 12- and 14-day-old embryos. **(A)** Representative agarose gels of the PCR products for H_1_R and GAPDH (internal control) from control E12 and E14 vMes tissue. –Ctl, negative control (500 ng total RNA without retrotranscription); +Ctl, positive control (500 ng cDNA from adult cerebral cortex). **(B)** Semi-quantitative densitometry analysis for H_1_R expression at E12 and E14. Values are expressed as percentage of the optic densities obtained at E12 and are means ± standard error (SEM) from eight experiments. **(C)** Specific [^3^H]-mepyramine binding to membranes. Values are expressed as fmol/mg protein, and are means ± SEM from five experiments. Membranes from adult cerebral cortex and human astrocytoma U373 MG cells were used as positive controls. *P*-values were obtained after two-tailed unpaired Student’s *t*-test. **(D)** H_1_R immunodetection. Left, coronal view of the mesencephalon at E12. The area outlined in red corresponds to the area shown in the representative epifluorescence micrographs (20×) on the right, which show independent and merged channels for H_1_Rs (red), Nestin (green), and DAPI (blue). MZ, marginal zone; VZ, ventricular zone; TNE, tegmental neuroepithelium; *varp*, aqueduct pretectal; *vat*, aqueduct tegmental; *vl*, ventricular lumen. Scale bar, 100 μm.

Semiquantitative PCR analysis of the expression of H_1_R in the vMes showed higher expression at E12 as compared with E14 (Figure 2A,B). The nucleotide BLAST on representative purified PCRs products from each embryo day and adult cerebral cortex yielded 100% identity with the rat H_1_R sequence (nucleotides 594–885 from NM_017018.1, data not shown).

Due to the high amount of tissue required for binding assays, [^3^H]-mepyramine binding was tested in a total membrane preparation from vMes (mesencephalon and telencephalon). Likewise, RT-PCR determinations, a higher density of [^3^H]-mepyramine binding sites was observed at E12 compared with E14 (Figure 2C; 142 ± 28 and 43 ± 5 fmol/mg protein), and at E12 the density of binding sites was even higher that in the cerebral cortex of adult rats and the U373 MG cell line (68 ± 7 and 118 ± 36 fmol/mg protein, respectively, five determinations), used as positive controls.

Immunofluorescence analysis showed that at E12 H_1_R^+^ cells were preferentially located in the ventricular zone (VZ), where NSCs are located (Figure 2D). Although, NSCs still showed H_1_R^+^ immunoreactivity at E14, specific labeling was observed outside VZ (Supplementary Figure S2B), indicating that other cell types, such as progenitors or neuroblasts, also express H_1_Rs at this embryo day (La Manno et al., 2016).

### Short- and Long-Term Effect of Chlorpheniramine on Dopaminergic Markers

The daily systemic administration of chlorpheniramine to pregnant rats during the differentiation and maturation stages of dopaminergic neurons (E12, E13, and E14) promoted contrasting short- and long-term effects on the development of the SNpc.

**FIGURE 3:**
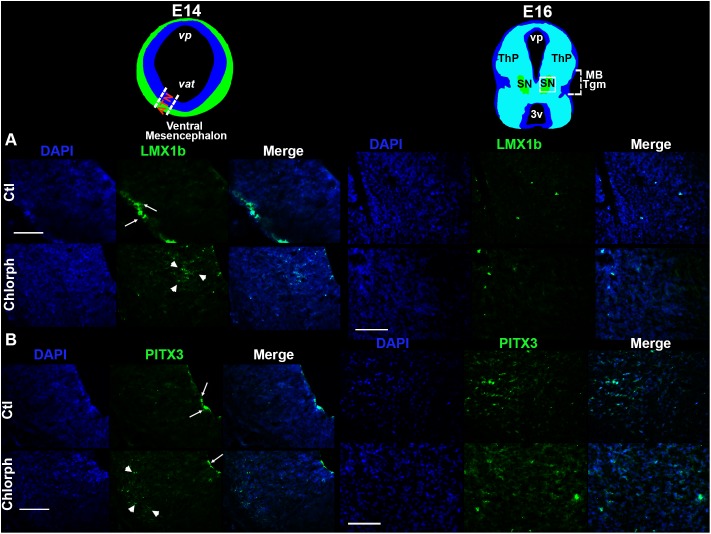
Effect of chlorpheniramine administration to pregnant rats on dopaminergic commitment and differentiation in the ventral mesencephalon of E14 and E16 embryos. Top panel, coronal views of the mesencephalon at E14 (in blue is the ventricular zone and in green the marginal zone or differentiated field) and E16 (substantia nigra in green). The outlined area in white correspond to the area where epifluorescence micrographs (20×) were taken. **(A,B)** Representative micrographs showing individual and merged channels for LMX1b immunoreactivity (green in **A**; arrows shown the mark in the marginal zone and arrow heads in the subventricular zone of the mesencephalic neuroepithelium) and PITX3 (green in **B**; arrows show the mark in the ventricular zone and arrow heads in the differentiation field of the mesencephalic neuroepithelium), and nuclei stained with DAPI in blue in coronal sections of E14 and E16 embryos from control (Ctl) and chlorpheniramine-treated (Chlorph) rats. ATN, anterior tegmental neuroepithelium; vp, aqueduct pretectal; *vat*, aqueduct tegmental; MB Tgm, Midbrain Tegmentum; SN, substantia nigra; ThP, thalamus posterior; 3v, third ventricle. Scale bar, 50 μm.

**FIGURE 4:**
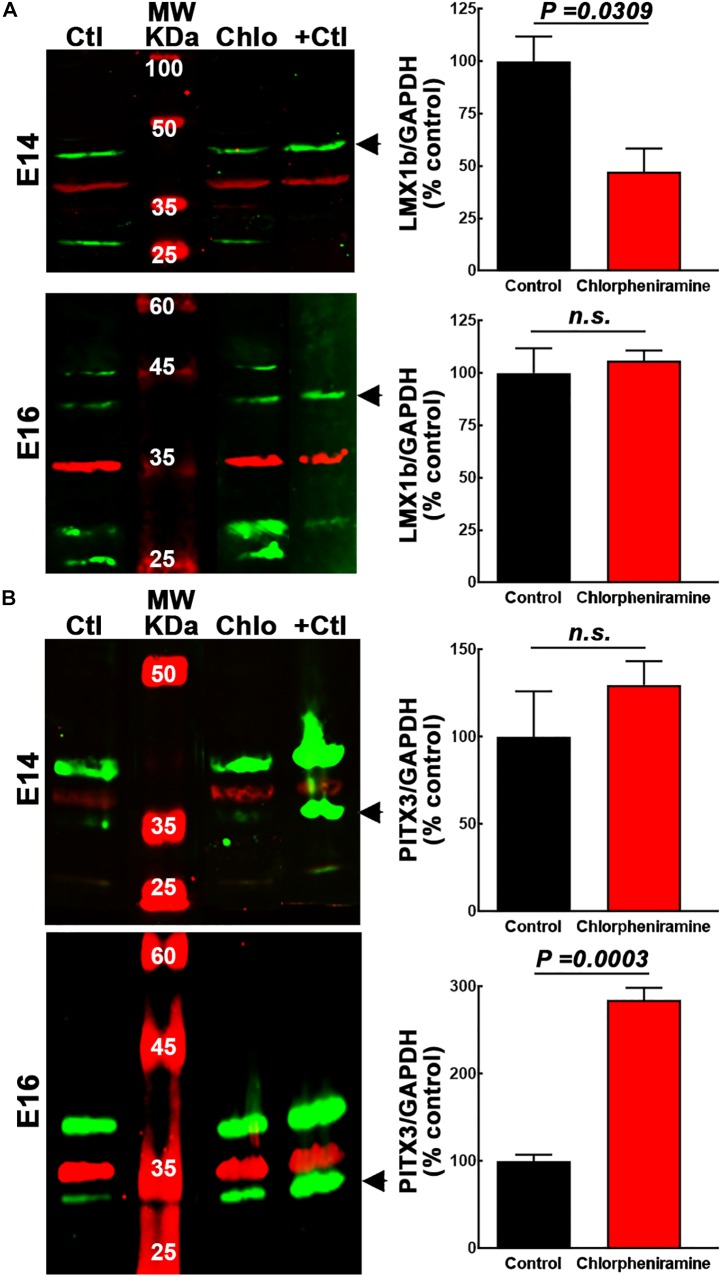
Quantitative analysis of the effect of chlorpheniramine administration to pregnant rats on dopaminergic commitment and differentiation in the ventral mesencephalon of 14- and 16-day-old embryos. Left columns show representative Western blots for LMX1b (green, 41 kDa) in panel **(A)** and PITX3 (green, 32 kDa) in panel **(B)**, for E14 and E16 ventral mesencephalon (vMes) from embryos from control (Ctl) and chlorpheniramine-treated (Chlo) rats. The internal control (GAPDH, 37 kDa) appears in red. Protein extracts from vMes E12 or adult substantia nigra were used as positive controls (+Ctl) for LMX1b and PITX3, respectively. MW, molecular weight ladder in kDa. Right columns, semi-quantitative fluorometry analysis of LMX1b **(A)** and PITX3 **(B)**, for E14 and E16 tissues from the vMes. Values are expressed as percentage of the fluorescence ratio of the control tissues and are means ± SEM from four experiments. *P*-values were obtained after two-tailed unpaired Student’s *t*-test; *n.s.*, non-significant.

Immunofluorescence showed that LMX1b was expressed by cells in the marginal zone (MZ) in control E14 vMes, while in the chlorpheniramine group this marker was observed in the subventricular zone (SVZ; Figure 3A). As expected, 48 h later LMX1b expression was markedly reduced to become practically undetected by immunofluorescence. In accord with this result, the semi-quantitative analysis of the protein levels showed a significant decrease at E14 in embryos from chlorpheniramine-treated rats compared with the control group (Figure 4A).

**FIGURE 5:**
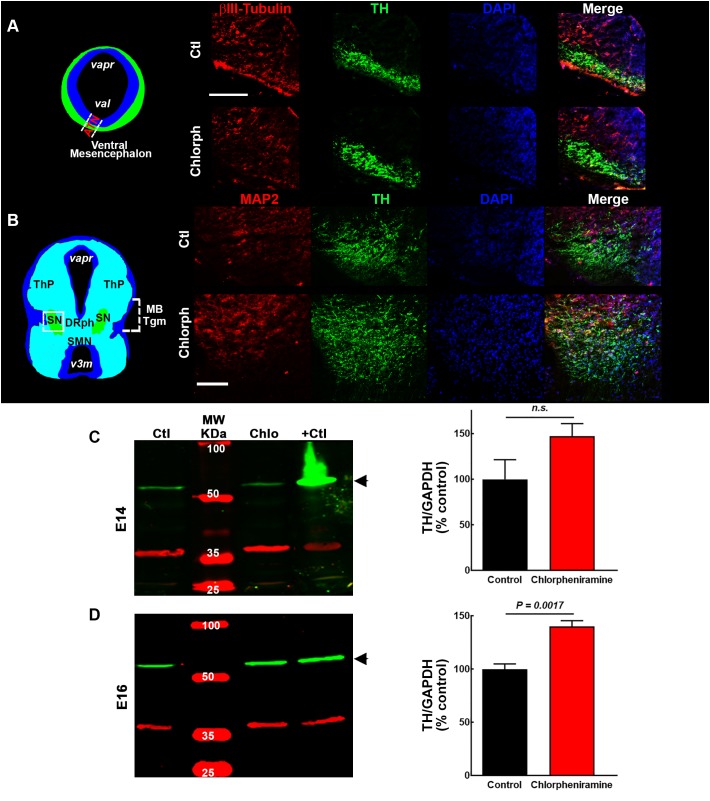
Effect of chlorpheniramine administration to pregnant rats on tyrosine hydroxylase (TH) immunoreactivity and protein levels in the ventral mesencephalon of 14- and 16-day-old embryos. **(A,B)** Left, coronal view of the mesencephalon at E14 and E16, respectively. The area outlined in white dashed lines and square corresponds to the area shown in the representative epifluorescence micrographs (20×) on the right, which show independent and merged channels for βIII-tubulin **(A)** and MAP2 **(B)** in red, TH in green, and DAPI (blue) for the vMes from embryos from control (Ctl) and chlorpheniramine-treated (Chlorph) rats. ATM, anterior tegmental neuroepithelium; *varp*, aqueduct pretectal; *vat*, aqueduct tegmental, MBTgm, Midbrain Tegmentum; SN, substantia nigra; ThP, thalamus posterior; DRph, dorsal raphe; SMN, supramammillary nucleus; *v3m*, third ventricle. Scale bar, 50 μm. **(C,D)** Left columns, representative Western blots for TH (green, 60 kDa) and the internal control GAPDH (red, 37 kDa), for the vMes from embryos from control (Ctl) and chlorpheniramine-treated (Chlo) rats. Protein extracts from adult substantia nigra were used as positive controls (+Ctl). MW, molecular weight ladder (kDa). Right column, quantitative fluorometry analysis of TH at E14 **(C)** and E16 **(D)**. Values are expressed as percentage of the fluorescence ratio of the control tissues and are means ± SEM from four experiments. *P*-values were obtained after two-tailed unpaired Student’s *t*-test; *n.s.*, non-significant.

Regarding, the dopaminergic markers in control embryos at E14, the immunofluorescence showed scarce and strong staining, for PITX3 and TH, respectively (Figure 3B, 5A,B). As expected, at E16 both markers were clearly distinguishable (Figure 3B, 5A,B), and an increase in the PITX3 and TH immunoreactivities was observed in the developing SNpc in chlorpheniramine group as compared with control animals (Figure 3B, 5B). The increased expression of dopaminergic markers was confirmed by the Western Bolt analysis, as a significant increase in the chlorpheniramine group was observed for both proteins at E16 (Figure 4B, 5D) and no significant changes were observed at E14 (Figure 4B, 5C).

**FIGURE 6:**
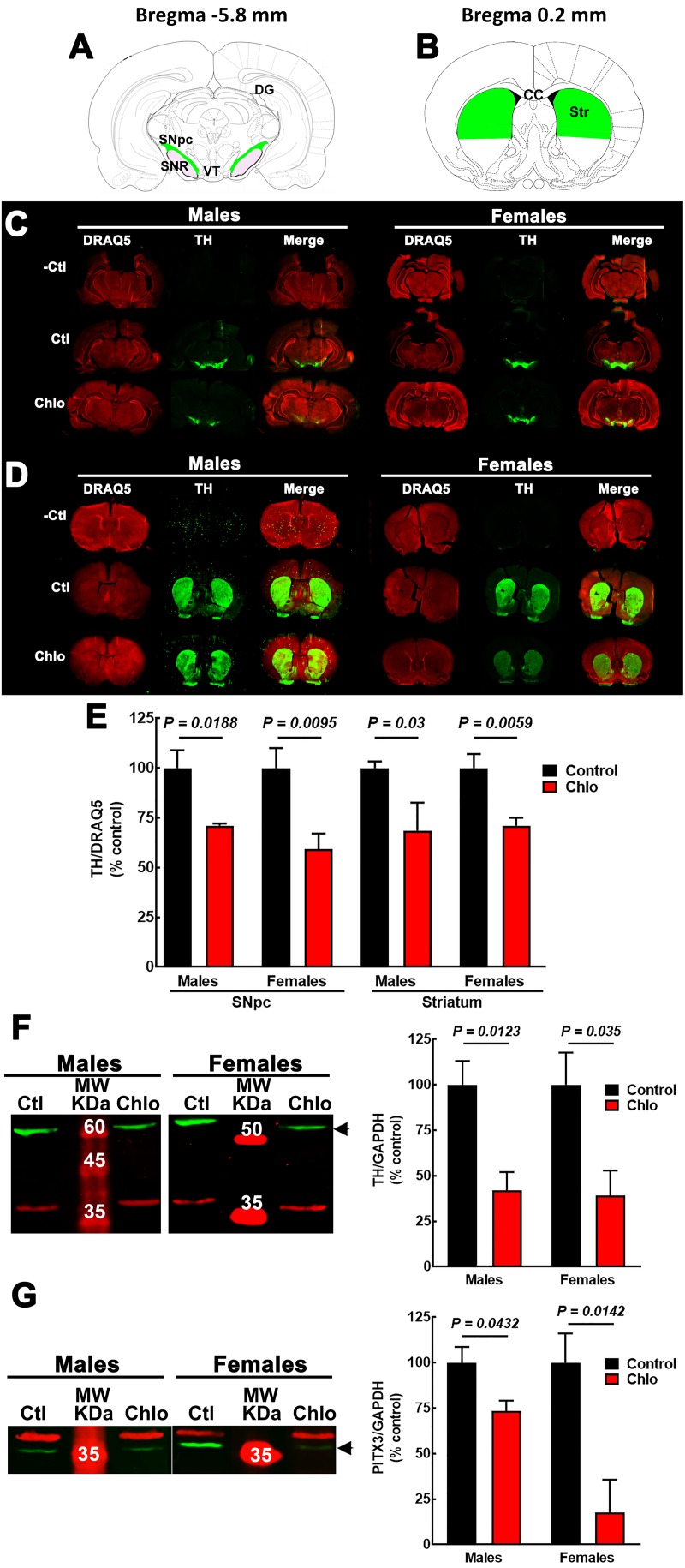
Effect of chlorpheniramine administration to pregnant rats on tyrosine hydroxylase (TH) immunoreactivity and protein levels in the substantia nigra and striatum from 21-day-old offsprings. **(A,B)** Cartoons of coronals sections at Bregma –5.8 and 0.2 mm. The areas in green were the anatomical region determined for TH immunoreactivity quantification in substantia nigra *pars compacta* (**A**, SNpc) and striatum (**B**, Str). Modify from Paxinos and Watson. **(C,D)** Representative images for TH immunoreactivity in brain coronal sections containing the SNpc **(C)** and the striatum **(D)** from 21-day-old male and female offsprings of control and chlorpheniramine-treated (Chlo) rats. Single and merged channels for the internal control DRAQ5 (red, nuclei) and TH (green) are shown. –Ctl, negative control (incubation without the primary antibody); Ctl, control, and Chlo, chlorpheniramine. **(E)** Quantitative analysis of TH immunoreactivity in SNpc and striatum of the offsprings from control and chlorpheniramine-treated (Chlo) rats. Values are expressed as percentage of the fluorescence ratio of the control tissues and are means ± SEM from four experiments. **(F,G)** Left, representative Western blots for the SNpc of male and female P21 offsprings from control (Ctl) and Chlo rats. The green band corresponds to TH (60 kDa) in panel **(F)** and to PITX3 (32 kDa) in panel **(G)**. For both panels, the red band corresponds to the internal control (GAPDH, 37 kDa). MW, molecular weight ladder (kDa). Right, quantitative fluorometry analysis of TH and PITX3. Values are expressed as percentage of the fluorescence ratio of the control tissues and are means ± SEM from four experiments. *P*-values were obtained after two-tailed unpaired Student’s *t*-test; *n.s.*, non-significant.

**FIGURE 7:**
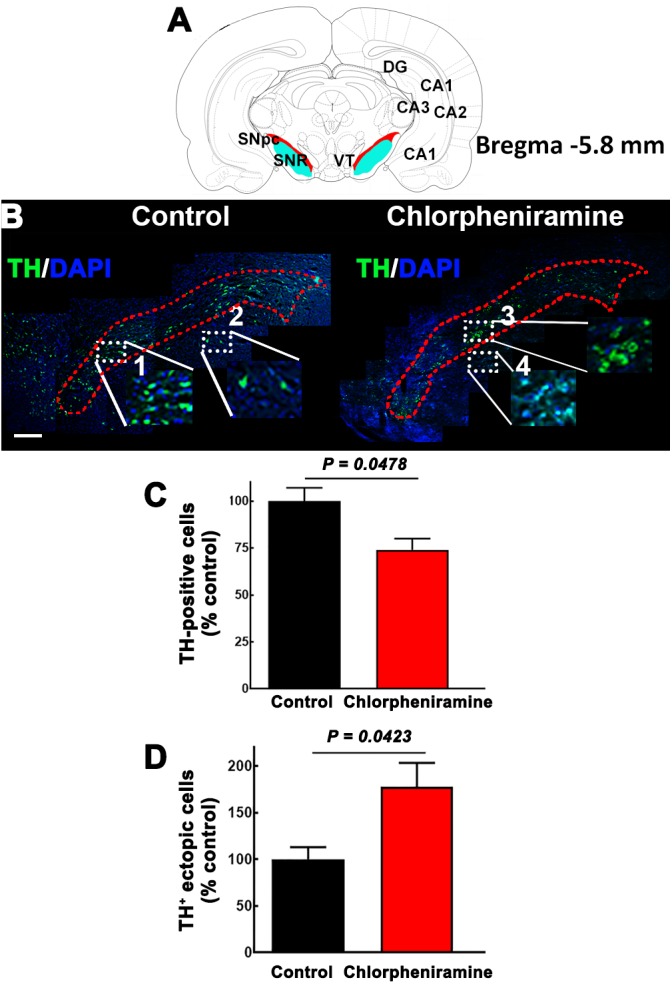
Effect of chlorpheniramine administration to pregnant rats on the number of tyrosine hydroxylase positive cells in the substantia nigra *pars compacta* of P21 pups. **(A)** Cartoon of a coronal section from Bregma –5.8 mm in red the substantia nigra *pars compacta*. Modify from Paxinos and Watson. **(B)** Merged representative epifluorescence micrographs (20×) for TH immunoreactivity (green) and DAPI (blue, nuclei) in the substantia nigra *pars compacta* (outlined in red dotted lines) of coronal brain sections (Bregma –5.8 mm) from pups from control and chlorpheniramine-treated rats. The white dotted rectangles represent digitalized zoomed areas (3.5×) from control (1, substantia nigra and 2, ectopic mark), and chlorpheniramine (3, substantia nigra and 4, ectopic mark) TH^+^ cells. Scale bar, 200 μm. **(C,D)** Quantification of TH^+^ cells. Values are expressed as percentage of the mean of total TH^+^ cells of the control, and are means ± SEM from three experiments (three consecutive sections were used per experiment). *P*-values were obtained after two-tailed unpaired Student’s *t*-test. SNpc, substantia nigra *pars compacta*; VT, Ventral tegmental area; SNR, substantia nigra reticulata; DG, dentate gyrus; Cornu Ammonis areas, CA1, CA2, and CA3.

Interestingly, at P21 quantitative immunofluorescence showed a significant decrease in the TH signal in the SNpc and dorsal striatum in both male and female pups from chlorpheniramine-treated rats as compared with control offspring (Figure 6A–E). Western blot analysis confirmed a lower expression of TH and PITX3 for both genders in the SNpc of the chlorpheniramine group as compared with controls (Figure 6F,G). Moreover, a reduction of 26 ± 6% in the number of TH^+^ cells was observed in the SNpc of chlorpheniramine-treated offspring compared with control animals (control, 205.5 ± 14.04 vs. chlorpheniramine, 152.8 ± 9.17; unilateral number of TH^+^ total cells; Figure 7). In both groups, TH^+^ cells were observed out and nearby of SNpc, however, P21 pups from chlorpheniramine-treated rats presented a significant increase in the number of ectopic TH^+^ cells (control, 13.08 ± 1.71 vs. chlorpheniramine, 23.06 ± 5.92; TH^+^ ectopic cells Figure 7).

### Striatal Dopamine Content and Release in P21 Pups

**FIGURE 8:**
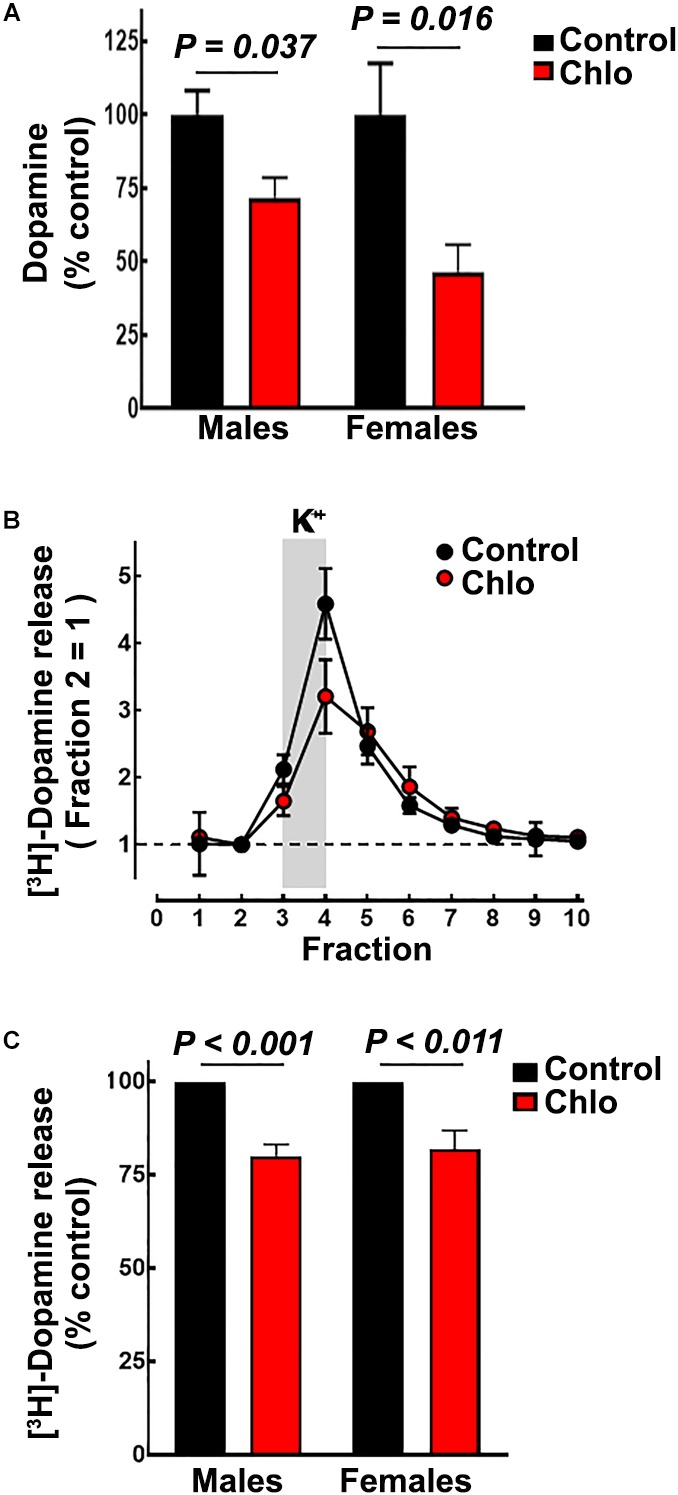
Effect of chlorpheniramine administration to pregnant rats on striatal dopamine content and release in 21-day-old offsprings. **(A)** Striatal dopamine content in male and females P21 offspring from control and chlorpheniramine-treated (Chlo) rats. Values are expressed as percentage of dopamine content of the control animals and are means ± SEM from 4–6 determinations from individual rats. **(B)** Representative experiment for depolarization-evoked [^3^H]-dopamine release from striatal slices of male P21 offspring from control (black circles) and chlo-treated rats (red circles). [^3^H]-dopamine release was evoked by raising the K^+^ concentration from 4 to 30 mM for the period indicated by the vertical gray bar. Values were the normalized to the [^3^H]-dopamine release of fraction 2 = 1, and represent means ± SEM from three replicates. **(C)** Analysis of the areas under the curve, after subtraction of basal [^3^H]-dopamine release. Values are expressed as a percentage of the release from control animal slices and are means ± SEM from 4 or 6 experiments for females or males, respectively. *P*-values were obtained after two-tailed unpaired Student’s *t*-test.

In accordance with the reduction in TH innervation to the striatum (Figure 8B,C), dopamine content was significantly lower in male and female pups from chlorpheniramine-treated animals (Figure 8A; 71.7 ± 6.9 and 42.9 ± 9% of control values, respectively).

Depolarization-evoked [^3^H]-dopamine release from striatal slices was markedly dependent on the presence of Ca^2+^ ions in the perfusion medium (91.8 ± 1.2% of total release, *P* < 0.001, Student’s *t*-test; four experiments; Supplementary Figure S3). Depolarization-induced [^3^H]-dopamine release was significantly lower in slices from pups from chlorpheniramine-treated animals compared with slices from control pups (Figure 8B,C; 80.05 ± 3.13 and 81.78 ± 5.08% of control release for male and female pups, respectively).

The reduction in [^3^H]-dopamine release observed for striatal slices from pups from chlorpheniramine-treated rats could be explained by over-expression of D_2_ autoreceptors on dopaminergic nerve terminals. However, the analysis of D_2_ receptor density in synaptosomal membranes showed no significant differences between groups (Supplementary Figure S4).

### Motor Activity in P21 Pups

**FIGURE 9:**
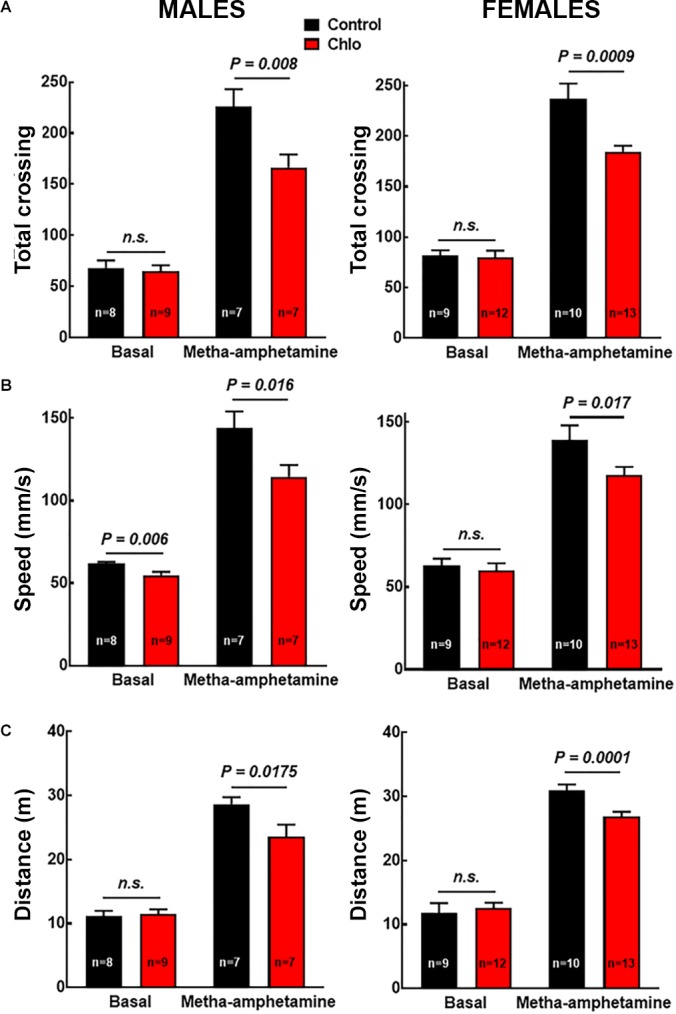
Effect of chlorpheniramine administration to pregnant rats on the motor activity of 21-day-old offsprings. **(A)** Total crossings; **(B)** speed; and **(C)** distance traveled. Evaluated in the open field test under basal conditions and 30 min after the administration of meta-amphetamine (1 mg/kg, i.p.) in male and female P21 offsprings from control and chlorpheniramine-treated (Chlo) rats. Values are means ± SEM from the indicated number of animals. *P*-values were obtained after two-tailed unpaired Student’s *t*-test; *n.s.*, non-significant.

To evaluate the possible functional implications of the reduction in TH innervation and dopamine release, motor activity in the open field test was evaluated under basal conditions and in response to the systemic administration of methamphetamine (1 mg/kg, i.p.). No changes in basal motor activity were observed between pups from control and chlorpheniramine-treated rats, except for a significant reduction in speed in male pups. In contrast, for methamphetamine-induced motor activity both genders of the chlorpheniramine-treated group showed a significant decrease in total crossings, speed and traveled distance in comparison with the control group (Figure 9).

## Discussion

In this study, we show that the systemic administration of the H_1_R antagonist/inverse agonist chlorpheniramine to pregnant rats at the embryonic days E14–E16 favors the differentiation of dopaminergic neurons in the embryonic brain, but hampers dopaminergic innervation to the striatum in P21 offsprings, indicating a role for H_1_Rs in vMes dopaminergic development.

Chlorpheniramine also acts as antagonist at all five muscarinic receptors (M_1_–M_5_), although the IC_50_ values for the inhibition of [^3^H]-N-methyl scopolamine binding to recombinant receptors (17–78 μM) indicates low affinity binding (Yasuda and Yasuda, 1999), in contrast with the high affinity of H_1_Rs for chlorpheniramine, with inhibition constant (K_i_) 1–3 nM (Arias-Montaño and Young, 1993; Hishinuma et al., 2017). Furthermore, in the rat mesencephalon muscarinic M_1_ and M_2_ receptors are expressed from E16 (Schlumpf et al., 1991), supporting that the effects of chlorpheniramine reported here are mediated by H_1_Rs.

H_1_Rs couple to Gα_q/11_ proteins and therefore their activation stimulates phospholipase C to produce the second messengers inositol-1,4,5-trisphosphate (IP_3_) and diacylglycerol (Hill et al., 1997; Panula et al., 2015). IP_3_ increases the intracellular concentration of calcium ions ([Ca^2+^]_i_) upon the activation of receptors present in the endoplasmic reticulum, whereas diacylglycerol activates the classical and novel classes of protein kinase C, PKC (Steinberg, 2008; Zeng et al., 2012). H_1_R activation increases the [Ca^2+^]_i_ in both cortical and vMes NSCs/NPCs (Molina-Hernández et al., 2013; Escobedo-Ávila et al., 2014), and changes in the [Ca^2+^]_i_ participate in a temporal- and spatial-dependent manner in the proliferation and differentiation of NSCs/NPCs (Somasundaram et al., 2014; Louhivuori et al., 2018), and are also a critical component in ontogenesis contributing to the formation and maintenance of dendritic structures (Lohmann et al., 2002; Lohmann and Wong, 2005).

In agreement with the immunohistochemistry and RT-PCR analysis of E12 and E14 vMes tissues, [^3^H]-mepyramine binding showed a higher density of H_1_Rs at E12 compared with E14 in membranes from the mesencephalon/telencephalon (3.3-fold; Figure 2C). The *in vivo* effects of chlorpheniramine could be due to the antagonism of endogenous histamine, although the high expression of H_1_Rs at E12 may also imply receptor constitutive activity, as reported in heterologous systems and in condition such allergies, where the receptor is highly expressed (Bakker et al., 2000; Nijmeijer et al., 2010). In this regard, Vanhala et al. (1994) reported that at E12 the histamine content in the mesencephalon (∼0.08 pmol/mg protein) is approximately sixfold lower that at E14. The possibility of constitutive activity of this receptor during CNS development has been previously reported by our group under *in vivo* physiological and pathological conditions for cortical development (Molina-Hernández and Velasco, 2008; Solís et al., 2017), although more experiments should be done in order to ensure that this phenomenon is taking place during CNS development, this concept could be further supported if, a single administration of the antihistamine drug at E12 could increase DAn markers. Although a formal quantification was not performed with a single administration of chlorpheniramine (E12), supplementary immunofluorescence for TH in the vMes presented here as Supplementary Figure S5 further suggest constitutive activity, as an increase in vMes TH immunoreactivity at E16 can be observed under this condition versus its corresponding control group. The above, is in accordance with higher density of H_1_R at E12 compared to E14 (Figure 1).

The VZ location of H_1_R immunoreactivity in the vMes neuroepithelium at E12, suggests that the effect of chlorpheniramine is mainly exerted at H_1_Rs expressed in NSCs/NPCs, whereas the reduction in LMX1b alongside the increase in PITX3 and TH immunoreactivity suggest that H_1_R-expressing NPCs are undergoing increased dopaminergic differentiation/maturation and impaired migration, with the latter effect possibly mediated by changes in [Ca^2+^]_i_ that lead to the differentiation of NSCs/NPCs.

The short-term effect reported here differs from those reported in the cerebral cortex neuroepithelium, where chlorpheniramine reduced neuron and deep layer glutamatergic differentiation at E12 (Molina-Hernández et al., 2013). The latter results are in accord with the reported effect of Ca^2+^ ions on cell differentiation, since an increase in Ca^2+^ signaling slows the cell cycle and increases neurogenesis (Berridge et al., 2000; Stanslowsky et al., 2018). However, it has been reported that a reduction in the [Ca^2+^]_i_ may also increase cell proliferation and differentiation (Toth et al., 2016), which is in accordance with our results that show increased differentiation of dopaminergic neurons in the vMes of the offsprings from chlorpheniramine-treated rats. The distinct effects could be explained by the expression of different Ca^2+^ “*tool kits*” by cells from the cortical and mesencephalic neuroepithelia, such as endoplasmic receptors (IP_3_ or ryanodine receptors), channels mediating store-activated Ca^2+^ entry (SOCE), Ca^2+^-sensitive channels, cytoplasmic Ca^2+^ binding proteins and Ca^2+^ pumps.

Differences in histamine-induced Ca^2+^ transients between cortical and vMes NSCs have been reported. Whereas in cortical NSCs 100 μM histamine increases only 2.2-fold the [Ca^2+^]_i_ (Escobedo-Ávila et al., 2014), in vMes NSCs a lower concentration (10 μM) promotes a fourfold increase in the [Ca^2+^]_i_ (Molina-Hernández et al., 2013). These data suggest important differences in the mechanisms involved in histamine-evoked Ca^2+^ transients and Ca^2+^ homeostasis. Store-operated Ca^2+^ entry (SOCE) is the main source of extracellular Ca^2+^ in NSCs/NPCs, and differential roles during development depending on the type of SOCE present have been suggested, although the regional identity of the channels mediating Ca^2+^ entry in the developing nervous system has not been defined (Morgan et al., 2013; Somasundaram et al., 2014; Stanslowsky et al., 2018).

The results observed in chlorpheniramine-treated rats’ pup’s (P21) on striatal dopaminergic innervation, the number of TH^+^ neurons in the SNpc, depolarization-induced dopamine release, and methamphetamine stimulated motor activity, indicates impaired dopaminergic transmission.

Dopamine synthesis and release are finely tuned by D_2_ auto-receptors located on the dopaminergic nerve terminals (Anzalone et al., 2012; Ford, 2014). However, at P21 there was no difference in D_2_ receptor density (Supplementary Figure S4), discarding this possibility to explain the observed reduction in depolarization-evoked dopamine release from striatal slices. We did not explore other components of dopaminergic homeostasis such as TH activity, dopamine re-uptake or its vesicular transport by the vesicular mono amine transporter 2 (VMAT2); however, one plausible explanation for our results is increased cell death before or during the complete maturation of the dopaminergic system. This notion is supported by the reduction in the number of TH^+^ cells in the SNpc found in pups from chlorpheniramine-treated rats, that could be explained by the inability of dopaminergic neurons to establish proper synaptic contacts, as the classic neurotrophic theory postulates (Burke, 2003). This concept is further supported by the presence of ectopic dopaminergic neurons in P21 pups from the chlorpheniramine group. However, aberrant dopaminergic innervation of these neurons to other brain regions cannot be discarded. Further research is required to establish the precise mechanisms underlying the reduced striatal dopaminergic innervations observed in post-natal life.

## Conclusion

Our data indicate that through the activation of H_1_Rs, histamine regulates the development of dopaminergic neurons in the vMes. This study may have clinical relevance since first-generation antihistamine drugs are still used during pregnancy (Kalpaklioglu and Baccioglu, 2012).

## Ethics Statement

All experiments and animal manipulation were in accordance to the Animal Welfare Assurance (A5281-01), “Guide for the Care and Use of Laboratory Animals” (NIH Publication No. 80-23, revised 1978) and “Norma Official Mexicana para la Producción, Cuidado y Uso de Animales de Laboratorio” (NOM-062-ZOO-1999). The protocol was approved by the Cinvestav Animal Care Committee and the Instituto Nacional de Perinatología Isidro Espinosa de los Reyes Research Animal Care (CICUAL), Biosecurity, and Ethic committees (protocol 3230-21202-01-2015).

## Author Contributions

All authors participated in the development of this research and drafting of the manuscript. BM-V, GA-M, and HP-Q performed the experiments and participated in the analysis of results. M-OQ-R participated in immunohistochemistry experiments and TH^+^ cells counting in P21 offspring. AB-N participated in the HPLC analysis. CL-R participated in motor activity experimental design and analysis. ND, J-AA-M, and AM-H contributed to the conception, critically revised the manuscript, supervised the experiments and data analysis, acquired funding, designed the experiments, and analyzed the data. AM-H approved the final version of the manuscript.

## Conflict of Interest Statement

The authors declare that the research was conducted in the absence of any commercial or financial relationships that could be construed as a potential conflict of interest.

## Footnotes

1 https://imagej.nih.gov/ij/

## Supplementary Material

FIGURE S1 LMX1b and PITX3 negative controls. Representative Western blots for LMX1b (green, 41 kDa) in the left A and PITX3 (green, 32 kDa) in the right, for E14 and E16 ventral mesencephalon (vMes) from embryos from Control and Chlorpheniramine-treated (Chlorph) rats. The internal control (GAPDH, 37 kDa) appears in red. Protein extracts from liver or spleen were used as negative control for LMX1b and PITX3, respectively. MW, molecular weight ladder in kDa.

FIGURE S2 H_1_R immunolocalization in the ventral mesencephalon in 14-day old embryos. **(A)** Coronal view of the mesencephalon at E14 showing the differentiation field (green) and the ventricular zone (blue). **(B)** Micrograph (20×) showing merged channels for H_1_Rs (red), Nestin (green), and DAPI (blue). VZ, ventricular zone; vat, aqueduct tegmental; vl, ventricular lumen. **(C)** Negative control for panel **(B)**. Scale bar, 100 μm.

FIGURE S3 Calcium dependence of the evoked released of Dopamine. **(A)** Representative experiment for depolarization-evoked [^3^H]-dopamine release from striatal slices of P21 offsprings from control rats. [^3^H]-dopamine release was evoked by raising the K^+^ concentration from 4 to 30 mM for the period indicated by the vertical gray bar. Values were the normalized to the [^3^H]-dopamine release of fraction 4 = 1, and represent means ± SEM from four replicates. **(B)** Quantitative analysis of the area under the curve after subtraction of the basal release. Values are expressed as a percentage of the release from slices exposed to Ca^2+^ and are means ± SEM from four experiments. *P*-values were obtained after two-tailed unpaired Student’s *t*-test.

FIGURE S4 Density of dopamine D_2_ receptors in membranes from striatal synaptosomes. Specific [^3^H]-spiperone binding expressed as fmol/mg protein in membranes synaptosomes from P21 pups from control or chlorpheniramine-treated (Chlo) treated rats. Values are means ± SEM from three independent experiments. *P*-values were obtained after two-tailed unpaired Student’s *t*-test; *n.s.*, non-significant.

FIGURE S5 Effect of a single injection of chlorpheniramine to pregnant rats at E12 on the ventral mesencephalon. **(A)** Coronal view of the mesencephalon at E16. The area outlined in white dashed square correspond to the area shown in the representative epifluorescence micrographs (20×) on the right. **(B)** Independent and merged channels for TH (green), MAP2 (red), and DAPI (blue) in embryos from control and chlorpheniramine-treated (Chlorph) pregnant rats. varp, aqueduct pretectal; SN, substantia nigra; ThP, thalamus posterior; DRph, dorsal raphe; SMN, supramammillary nucleus; v3m, third ventricle. Scale bar, 100 μm.

## Abbreviations

H_1_R: histamine H_1_ receptor
NPCs: neural progenitor cells
NSCs: neural stem cells
SNpc: substantia nigra *pars compacta*
TH: tyrosine hydroxylase
vMes: ventral mesencephalon

## References

[B1] Ambree O. Buschert J. Zhang W. Arolt V. Dere E. Zlomuzica A. (2014). Impaired spatial learning and reduced adult hippocampal neurogenesis in histamine H1-receptor knockout mice. *Eur. Neuropsychopharmacol.* 24 1394–1404. 10.1016/j.euroneuro.2014.04.006 24862254

[B2] Andersson E. Tryggvason U. Deng Q. Friling S. Alekseenko Z. Robert B. (2006). Identification of intrinsic determinants of midbrain dopamine neurons. *Cell* 124 393–405. 10.1016/j.cell.2005.10.037 16439212

[B3] Anzalone A. Lizardi-Ortiz J. E. Ramos M. De Mei C. Hopf F. W. Iaccarino C. (2012). Dual control of dopamine synthesis and release by presynaptic and postsynaptic dopamine D2 receptors. *J. Neurosci.* 32 9023–9034. 10.1523/JNEUROSCI.0918-12.2012 22745501 PMC3752062

[B4] Arias-Montaño J. A. Young J. M. (1993). Characteristics of histamine H1 receptors on HeLa cells. *Eur. J. Pharmacol.* 245 291–295. 10.1016/0922-4106(93)90110-U 8335064

[B5] Bakker R. A. Wieland K. Timmerman H. Leurs R. (2000). Constitutive activity of the histamine H(1) receptor reveals inverse agonism of histamine H(1) receptor antagonists. *Eur. J. Pharmacol.* 387 R5–R7. 10.1016/S0014-2999(99)00803-1 10633171

[B6] Bernardino L. Eiriz M. F. Santos T. Xapelli S. Grade S. Rosa A. I. (2012). Histamine stimulates neurogenesis in the rodent subventricular zone. *Stem Cells* 30 773–784. 10.1002/stem.1042 22893458

[B7] Berridge M. J. Lipp P. Bootman M. D. (2000). The versatility and universality of calcium signalling. *Nat. Rev. Mol. Cell Biol.* 1 11–21. 10.1038/35036035 11413485

[B8] Bradford M. M. (1976). A rapid and sensitive method for the quantitation of microgram quantities of protein utilizing the principle of protein-dye binding. *Anal. Biochem.* 72 248–254. 10.1016/0003-2697(76)90527-3 942051

[B9] Burke R. E. (2003). Postnatal developmental programmed cell death in dopamine neurons. *Ann. N. Y. Acad. Sci.* 991 69–79. 10.1111/j.1471-4159.2009.06101.x 12846975

[B10] Chepkova A. Yanovsky E. Parmentier R. Ohtsu H. Haas H. L. Lin J. S. (2012). Histamine receptor expression, hippocampal plasticity and ammonia in histidine decarboxylase knockout mice. *Cell Mol. Neurobiol.* 32 17–25. 10.1007/s10571-011-9730-1 21710252 PMC11498542

[B11] Escobedo-Ávila I. Vargas-Romero F. Molina-Hernández A. López-González R. Cortes D. De Carlos J. A. (2014). Histamine impairs midbrain dopaminergic development in vivo by activating histamine type 1 receptors. *Mol. Brain* 7:58. 10.1186/s13041-014-0058-x 25112718 PMC4237960

[B12] Ford C. P. (2014). The role of D2-autoreceptors in regulating dopamine neuron activity and transmission. *Neuroscience* 282 13–22. 10.1016/j.neuroscience.2014.01.025 24463000 PMC4108583

[B13] Gates M. A. Coupe V. M. Torres E. M. Fricker-Gates R. A. Dunnett S. B. (2004). Spatially and temporally restricted chemoattractive and chemorepulsive cues direct the formation of the nigro-striatal circuit. *Eur. J. Neurosci.* 19 831–844. 10.1111/j.1460-9568.2004.03213.x 15009130

[B14] Guerrero-Flores G. Bastidas-Ponce A. Collazo-Navarrete O. Guerra-Crespo M. Covarrubias L. (2017). Functional determination of the differentiation potential of ventral mesencephalic neural precursor cells during dopaminergic neurogenesis. *Dev. Biol.* 429 56–70. 10.1016/j.ydbio.2017.07.008 28733161

[B15] Hegarty S. V. Sullivan A. M. O’Keeffe G. W. (2013). Midbrain dopaminergic neurons: a review of the molecular circuitry that regulates their development. *Dev. Biol.* 379 123–138. 10.1016/j.ydbio.2013.04.014 23603197

[B16] Heron A. Rouleau A. Cochois V. Pillot C. Schwartz J. C. Arrang J. M. (2001). Expression analysis of the histamine H(3) receptor in developing rat tissues. *Mech. Dev.* 105 167–173. 10.1016/S0925-4773(01)00389-6 11429293

[B17] Hill S. J. Ganellin C. R. Timmerman H. Schwartz J. C. Shankley N. P. Young J. M. (1997). International union of pharmacology. XIII. classification of histamine receptors. *Pharmacol. Rev.* 49 253–278. 9311023

[B18] Hishinuma S. Kosaka K. Akatsu C. Uesawa Y. Fukui H. Shoji M. (2017). Asp73-dependent and -independent regulation of the affinity of ligands for human histamine H1 receptors by Na. *Biochem. Pharmacol.* 128 46–54. 10.1016/j.bcp.2016.12.021 28040476

[B19] Kalpaklioglu F. Baccioglu A. (2012). Efficacy and safety of H1-antihistamines: an update. *Antiinflamm. Antiallergy Agents Med. Chem.* 11 230–237. 10.2174/1871523011202030230 23173575

[B20] Karlstedt K. Senkas A. Ahman M. Panula P. (2001). Regional expression of the histamine H(2) receptor in adult and developing rat brain. *Neuroscience* 102 201–208. 10.1016/S0306-4522(00)00464-4 11226684

[B21] Kinnunen A. Lintunen M. Karlstedt K. Fukui H. Panula P. (1998). In situ detection of H1-receptor mRNA and absence of apoptosis in the transient histamine system of the embryonic rat brain. *J. Comp. Neurol.* 394 127–137. 10.1002/(SICI)1096-9861(19980427)394:1<127::AID-CNE10>3.0.CO;2-L 9550146

[B22] La Manno G. Gyllborg D. Codeluppi S. Nishimura K. Salto C. Zeisel A. (2016). Molecular diversity of midbrain development in mouse. human, and stem cells. *Cell* 167 566.e19–580.e19. 10.1016/j.cell.2016.09.027 27716510 PMC5055122

[B23] Lauder J. M. Bloom F. E. (1974). Ontogeny of monoamine neurons in the locus coeruleus, Raphe nuclei and substantia nigra of the rat. I. Cell differentiation. *J. Comp. Neurol.* 155 469–481. 10.1002/cne.901550407 4847734

[B24] Lohmann C. Myhr K. L. Wong R. O. (2002). Transmitter-evoked local calcium release stabilizes developing dendrites. *Nature* 418 177–181. 10.1038/nature00850 12110889

[B25] Lohmann C. Wong R. O. (2005). Regulation of dendritic growth and plasticity by local and global calcium dynamics. *Cell Calcium* 37 403–409. 10.1016/j.ceca.2005.01.008 15820387

[B26] Louhivuori L. M. Turunen P. M. Louhivuori V. Yellapragada V. Nordstrom T. Uhlen P. (2018). Regulation of radial glial process growth by glutamate via mGluR5/TRPC3 and neuregulin/ErbB4. *Glia* 66 94–107. 10.1002/glia.23230 28887860

[B27] Márquez-Gómez R. Robins M. T. Gutierrez-Rodelo C. Arias J. M. Olivares-Reyes J. A. van Rijn R. M. (2018). Functional histamine H3 and adenosine A2A receptor heteromers in recombinant cells and rat striatum. *Pharmacol. Res.* 129 515–525. 10.1016/j.phrs.2017.11.036 29217157 PMC6429923

[B28] Mobarakeh J. I. Takahashi K. Sakurada S. Nishino S. Watanabe H. Kato M. (2005). Enhanced antinociception by intracerebroventricularly administered orexin A in histamine H1 or H2 receptor gene knockout mice. *Pain* 118 254–262. 10.1016/j.pain.2005.08.024 16202530

[B29] Molina-Hernández A. Rodríguez-Martínez G. Escobedo-Ávila I. Velasco I. (2013). Histamine up-regulates fibroblast growth factor receptor 1 and increases FOXP2 neurons in cultured neural precursors by histamine type 1 receptor activation: conceivable role of histamine in neurogenesis during cortical development in vivo. *Neural Dev.* 8:4. 10.1186/1749-8104-8-4 23497494 PMC3601999

[B30] Molina-Hernández A. Velasco I. (2008). Histamine induces neural stem cell proliferation and neuronal differentiation by activation of distinct histamine receptors. *J. Neurochem.* 106 706–717. 10.1111/j.1471-4159.2008.05424.x 18419767

[B31] Morgan P. J. Hubner R. Rolfs A. Frech M. J. (2013). Spontaneous calcium transients in human neural progenitor cells mediated by transient receptor potential channels. *Stem Cells Dev.* 22 2477–2486. 10.1089/scd.2013.0061 23631375

[B32] Nijmeijer S. Leurs R. Vischer H. F. (2010). Constitutive activity of the histamine H(1) receptor. *Methods Enzymol.* 484 127–147. 10.1016/B978-0-12-381298-8.00007-1 21036230

[B33] Oo T. F. Burke R. E. (1997). The time course of developmental cell death in phenotypically defined dopaminergic neurons of the substantia nigra. *Brain Res. Dev. Brain Res.* 98 191–196. 10.1016/S0165-3806(96)00173-3 9051260

[B34] Panula P. Chazot P. L. Cowart M. Gutzmer R. Leurs R. Liu W. L. (2015). International union of basic and clinical pharmacology. XCVIII. histamine receptors. *Pharmacol. Rev.* 67 601–655. 10.1124/pr.114.010249 26084539 PMC4485016

[B35] Parmentier R. Zhao Y. Perier M. Akaoka H. Lintunen M. Hou Y. (2016). Role of histamine H1-receptor on behavioral states and wake maintenance during deficiency of a brain activating system: a study using a knockout mouse model. *Neuropharmacology* 106 20–34. 10.1016/j.neuropharm.2015.12.014 26723880

[B36] Paxinos G. Watson C. (2013). *The Rat Brain in Stereotaxic Coordinates.* 7th Edn Cambridge: Academic Press.

[B37] Rojas-Amigo A. (2014). Cálculo del tamaño muestral en procedimientos de experimentación con animales. valoración de las incidencias. *Animales de Laboratorio* 62 31–33.

[B38] Schlumpf M. Palacios J. M. Cortes R. Lichtensteiger W. (1991). Regional development of muscarinic cholinergic binding sites in the prenatal rat brain. *Neuroscience* 45 347–357. 10.1016/0306-4522(91)90232-D 1684836

[B39] Smidt M. P. Asbreuk C. H. Cox J. J. Chen H. Johnson R. L. Burbach J. P. (2000). A second independent pathway for development of mesencephalic dopaminergic neurons requires Lmx1b. *Nat. Neurosci.* 3 337–341. 10.1038/73902 10725922

[B40] Smidt M. P. Smits S. M. Burbach J. P. (2004). Homeobox gene Pitx3 and its role in the development of dopamine neurons of the substantia nigra. *Cell Tissue Res.* 318 35–43. 10.1007/s00441-004-0943-1 15300495

[B41] Solís K. H. Mendez L. I. Garcia-Lopez G. Diaz N. F. Portillo W. De Nova-Ocampo M. (2017). The histamine h1 receptor participates in the increased dorsal telencephalic neurogenesis in embryos from diabetic rats. *Front. Neurosci.* 11:676. 10.3389/fnins.2017.00676 29311766 PMC5735119

[B42] Somasundaram A. Shum A. K. McBride H. J. Kessler J. A. Feske S. Miller R. J. (2014). Store-operated CRAC channels regulate gene expression and proliferation in neural progenitor cells. *J. Neurosci.* 34 9107–9123. 10.1523/JNEUROSCI.0263-14.2014 24990931 PMC4078087

[B43] Soria-Jasso L. E. Bahena-Trujillo R. Arias-Montano J. A. (1997). Histamine H1 receptors and inositol phosphate formation in rat thalamus. *Neurosci. Lett.* 225 117–120. 10.1016/S0304-3940(97)00209-7Get 9147388

[B44] Specht L. A. Pickel V. M. Joh T. H. Reis D. J. (1981a). Light-microscopic immunocytochemical localization of tyrosine hydroxylase in prenatal rat brain. I. Early ontogeny. *J. Comp. Neurol.* 199 233–253. 10.1002/cne.901990207 6114114

[B45] Specht L. A. Pickel V. M. Joh T. H. Reis D. J. (1981b). Light-microscopic immunocytochemical localization of tyrosine hydroxylase in prenatal rat brain. II. Late ontogeny. *J. Comp. Neurol.* 199 255–276. 10.1002/cne.901990208 6114115

[B46] Stanslowsky N. Tharmarasa S. Staege S. Kalmbach N. Klietz M. Schwarz S. C. (2018). Calcium, sodium, and transient receptor potential channel expression in human fetal midbrain-derived neural progenitor cells. *Stem Cells Dev.* 27 976–984. 10.1089/scd.2017.0281 29779467

[B47] Steinberg S. (2008). Structural basis of protein kinase C isoform function. *Physiol. Rev.* 88 1341–1378. 10.1152/physrev.00034.2007 18923184 PMC2899688

[B48] Toth A. B. Shum A. K. Prakriya M. (2016). Regulation of neurogenesis by calcium signaling. *Cell Calcium* 59 124–134. 10.1016/j.ceca.2016.02.011 27020657 PMC5228525

[B49] Vanhala A. Yamatodani A. Panula P. (1994). Distribution of histamine-, 5-hydroxytryptamine-, and tyrosine hydroxylase-immunoreactive neurons and nerve fibers in developing rat brain. *J. Comp. Neurol.* 347 101–114. 10.1002/cne.903470108 7798375

[B50] Villanueva M. A. (2008). Electrotransfer of proteins in an environmentally friendly methanol-free transfer buffer. *Anal. Biochem.* 373 377–379. 10.1016/j.ab.2007.08.007 17850757

[B51] Vitalis T. Cases O. Parnavelas J. G. (2005). Development of the dopaminergic neurons in the rodent brainstem. *Exp. Neurol.* 191(Suppl. 1), S104–S112. 10.1016/j.expneurol.2004.05.044 15629757

[B52] Yan C. H. Levesque M. Claxton S. Johnson R. L. Ang S. L. (2011). Lmx1a and lmx1b function cooperatively to regulate proliferation, specification, and differentiation of midbrain dopaminergic progenitors. *J. Neurosci.* 31 12413–12425. 10.1523/JNEUROSCI.1077-11.2011 21880902 PMC6703256

[B53] Yasuda S. U. Yasuda R. P. (1999). Affinities of brompheniramine, chlorpheniramine, and terfenadine at the five human muscarinic cholinergic receptor subtypes. *Pharmacotherapy* 19 447–451. 10.1592/phco.19.6.447.31041 10212017

[B54] Zeng L. Webster S. V. Newton P. M. (2012). The biology of protein kinase C. *Adv. Exp. Med. Biol.* 740 639–661. 10.1007/978-94-007-2888-2_28 22453963

