# Supplementary figures and images for: The Systemic Administration of the Histamine H1 Receptor Antagonist/Inverse Agonist Chlorpheniramine to Pregnant Rats Impairs the Development of Nigro-Striatal Dopaminergic Neurons
Source: Front Neurosci. 2019 Apr 16;13:360. doi: 10.3389/fnins.2019.00360 (PMC6476962; doi:10.3389/fnins.2019.00360)

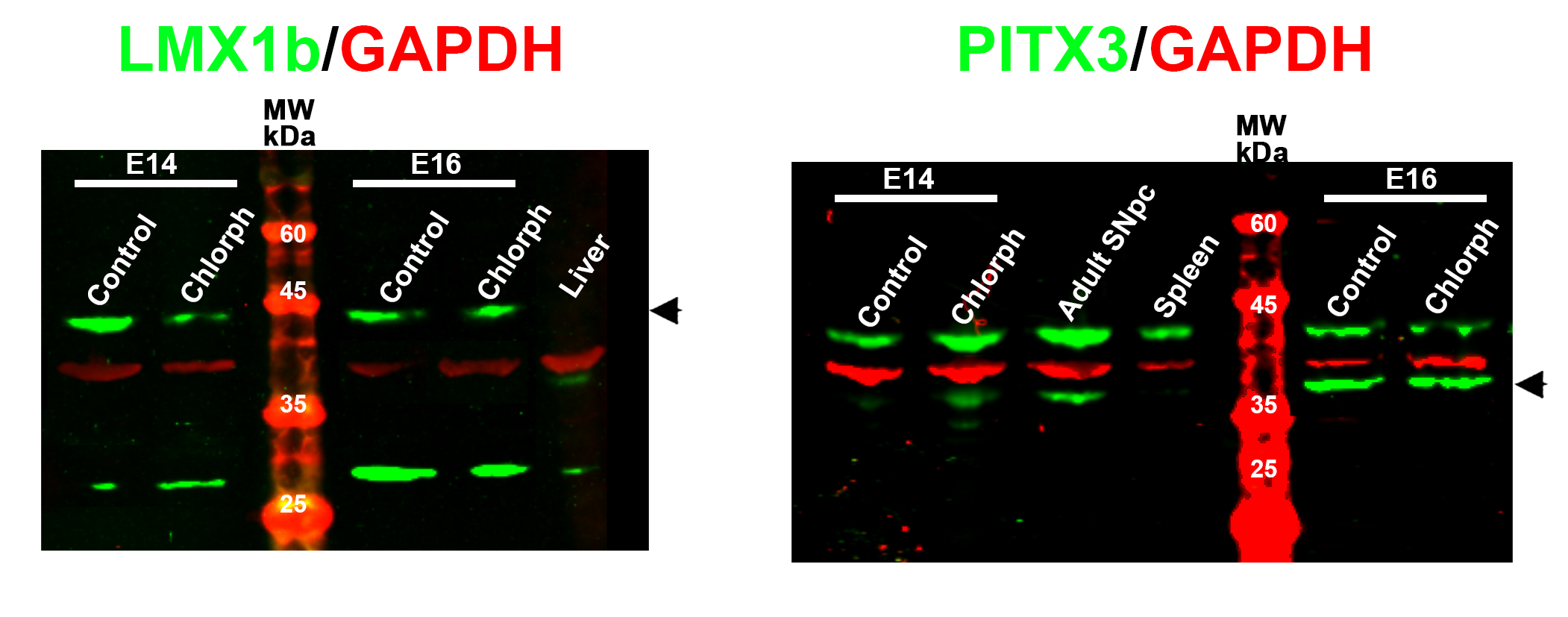

Supplement: FIGURE S1 — LMX1b and PITX3 negative controls. Representative Western blots for LMX1b (green, 41 kDa) in the left A and PITX3 (green, 32 kDa) in the right, for E14 and E16 ventral mesencephalon (vMes) from embryos from Control and Chlorpheniramine-treated (Chlorph) rats. The internal control (GAPDH, 37 kDa) appears in red. Protein extracts from liver or spleen were used as negative control for LMX1b and PITX3, respectively. MW, molecular weight ladder in kDa. [file Image_1.TIF]

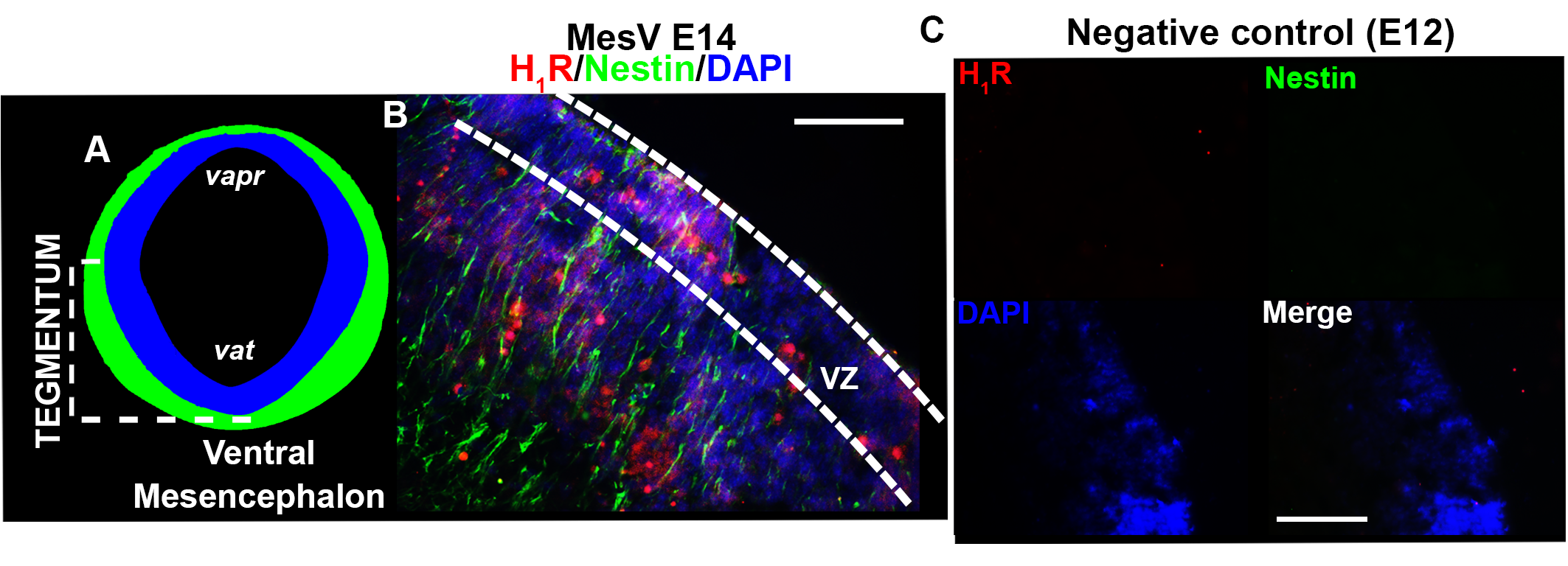

Supplement: FIGURE S2 — H1R immunolocalization in the ventral mesencephalon in 14-day old embryos. (A) Coronal view of the mesencephalon at E14 showing the differentiation field (green) and the ventricular zone (blue). (B) Micrograph (20×) showing merged channels for H1Rs (red), Nestin (green), and DAPI (blue). VZ, ventricular zone; vat, aqueduct tegmental; vl, ventricular lumen. (C) Negative control for panel (B). Scale bar, 100 μm. [file Image_2.TIF]

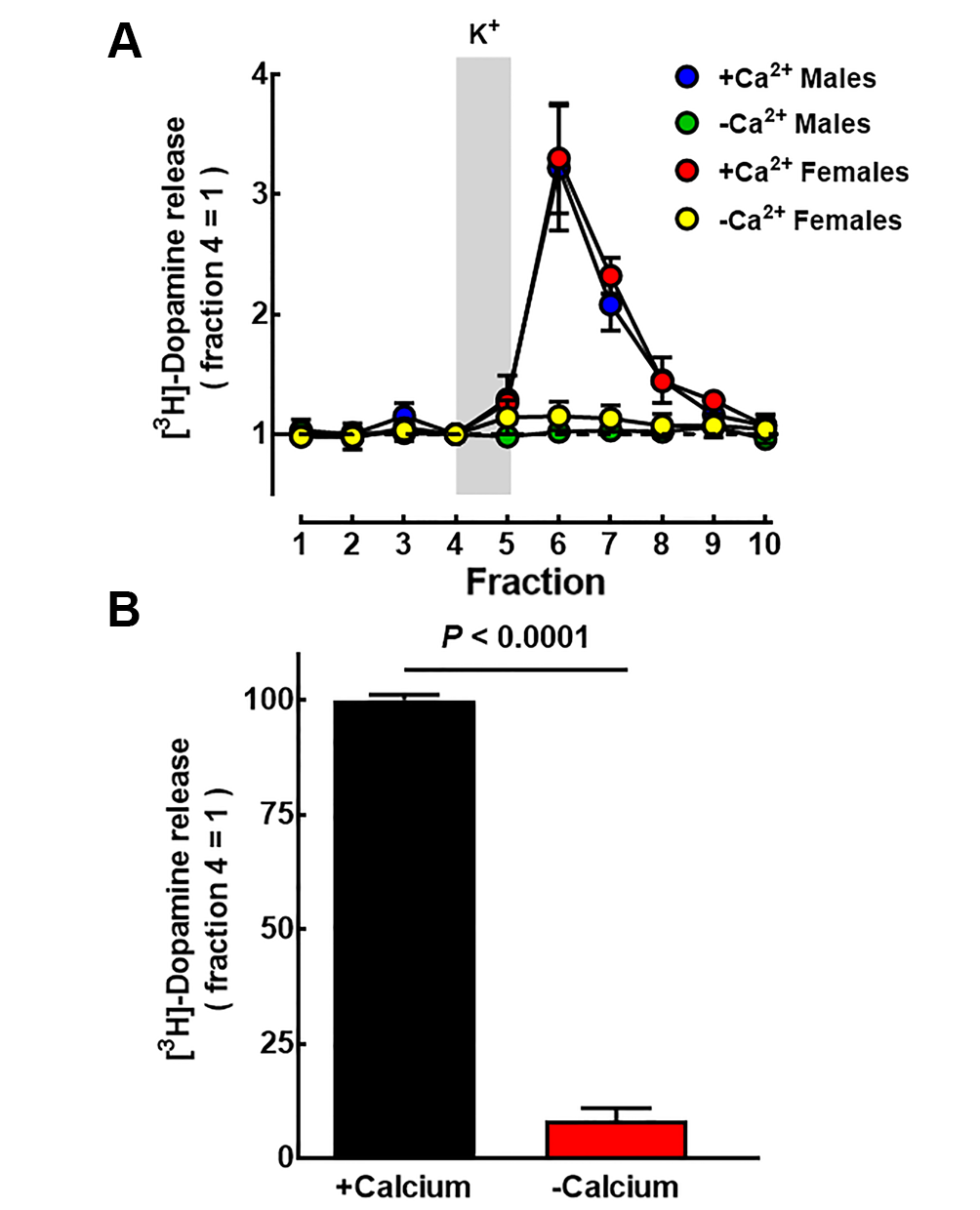

Supplement: FIGURE S3 — Calcium dependence of the evoked released of Dopamine. (A) Representative experiment for depolarization-evoked [3H]-dopamine release from striatal slices of P21 offsprings from control rats. [3H]-dopamine release was evoked by raising the K+ concentration from 4 to 30 mM for the period indicated by the vertical gray bar. Values were the normalized to the [3H]-dopamine release of fraction 4 = 1, and represent means ± SEM from four replicates. (B) Quantitative analysis of the area under the curve after subtraction of the basal release. Values are expressed as a percentage of the release from slices exposed to Ca2+ and are means ± SEM from four experiments. P-values were obtained after two-tailed unpaired Student’s t-test. [file Image_3.TIF]

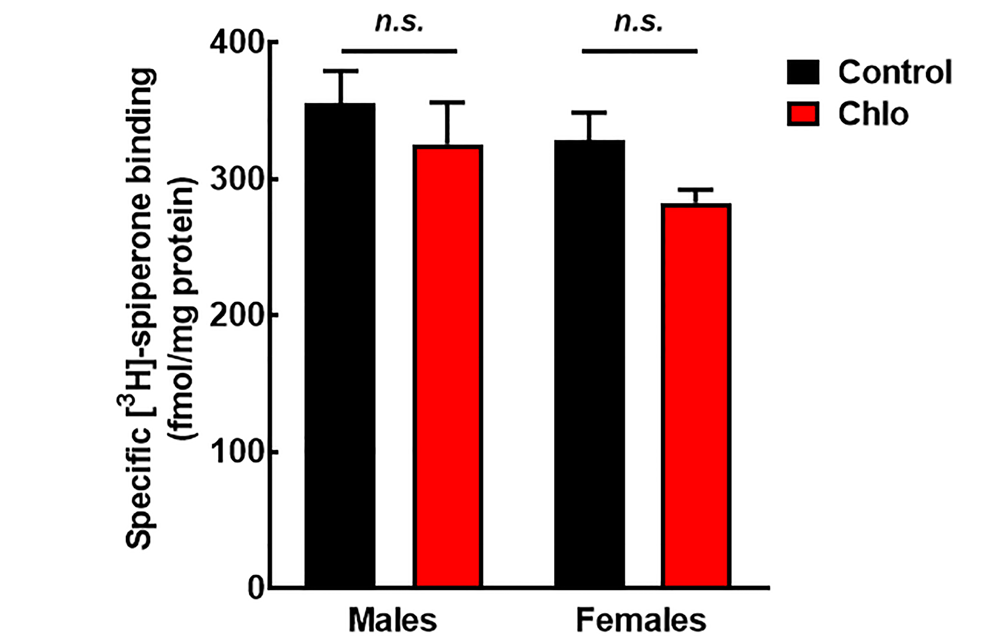

Supplement: FIGURE S4 — Density of dopamine D2 receptors in membranes from striatal synaptosomes. Specific [3H]-spiperone binding expressed as fmol/mg protein in membranes synaptosomes from P21 pups from control or chlorpheniramine-treated (Chlo) treated rats. Values are means ± SEM from three independent experiments. P-values were obtained after two-tailed unpaired Student’s t-test; n.s., non-significant. [file Image_4.TIF]

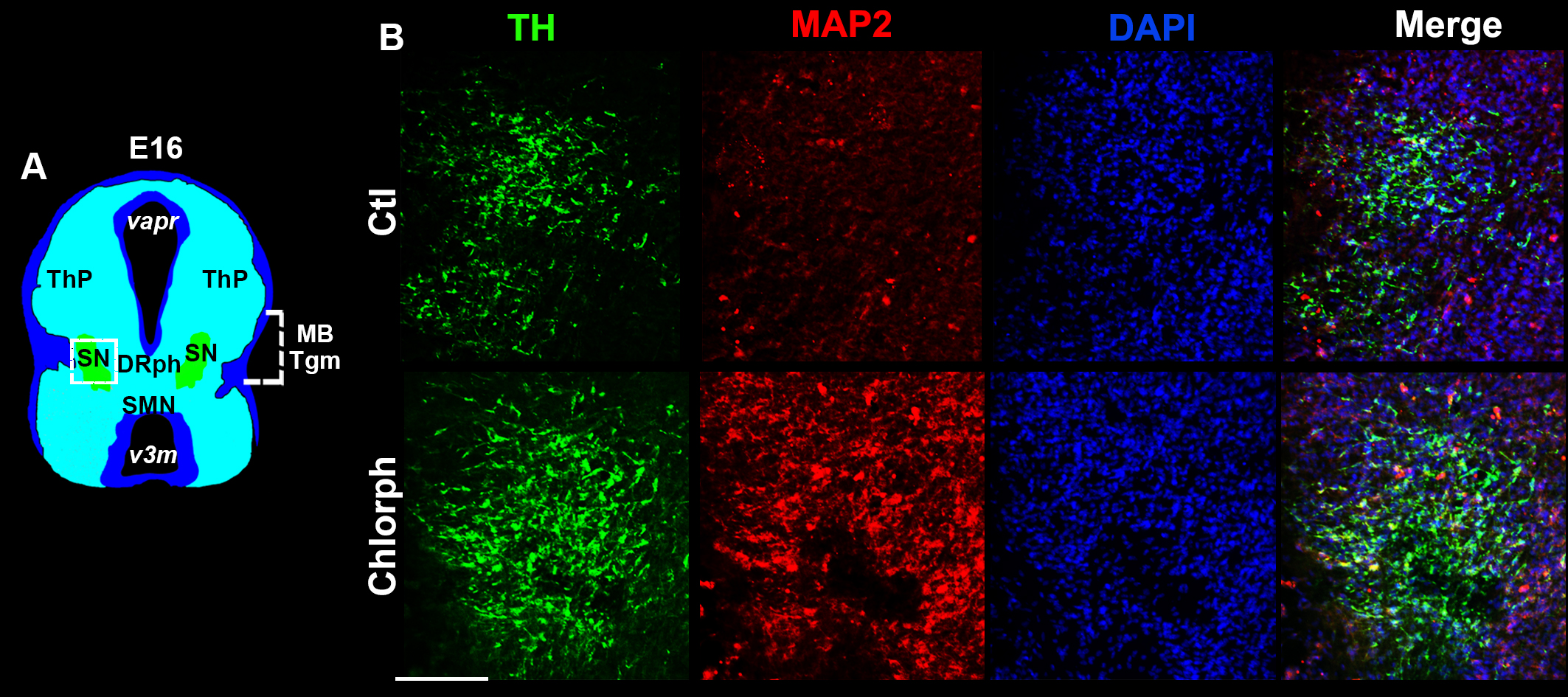

Supplement: FIGURE S5 — Effect of a single injection of chlorpheniramine to pregnant rats at E12 on the ventral mesencephalon. (A) Coronal view of the mesencephalon at E16. The area outlined in white dashed square correspond to the area shown in the representative epifluorescence micrographs (20×) on the right. (B) Independent and merged channels for TH (green), MAP2 (red), and DAPI (blue) in embryos from control and chlorpheniramine-treated (Chlorph) pregnant rats. varp, aqueduct pretectal; SN, substantia nigra; ThP, thalamus posterior; DRph, dorsal raphe; SMN, supramammillary nucleus; v3m, third ventricle. Scale bar, 100 μm. [file Image_5.JPEG]
